# Ethyl 2-isopropyl­amino-5-methyl-4-oxo-3-phenyl-3,4-dihydro­thieno[2,3-*d*]pyrimidine-6-carboxyl­ate

**DOI:** 10.1107/S1600536809045449

**Published:** 2009-11-04

**Authors:** Ai-Hua Zheng, Liang-Yong Huang, E. Chen, Hong Luo

**Affiliations:** a Institute of Medicinal Chemistry, Yunyang Medical College, Shiyan 442000, People’s Republic of China; b Department of Pharmacy, Taihe Hospital of Yunyang Medical College, Shiyan 442000, People’s Republic of China; c Department of Pharmacy, Renmin Hospital of Yunyang Medical College, Shiyan 442000, People’s Republic of China; d Department of Medicinal Physics, Yunyang Medical College, Shiyan 442000, People’s Republic of China

## Abstract

The title compound, C_19_H_21_N_3_O_3_S, was synthesized *via* an aza-Wittig reaction of a functionalized imino­phospho­rane with phenyl isocyanate under mild conditions. In the mol­ecule, the fused thienopyrimidine ring system makes a dihedral angle of 66.30 (11)° with the phenyl ring. An intra­molecular C—H⋯O hydrogen bond occurs. The terminal –OCH_2_CH_3_ group is disordered over two sites with refined occupancies of 0.537 (13) and 0.463 (13). The crystal packing is stabilized by inter­molecular C—H⋯O and N—H⋯O hydrogen bonds.

## Related literature

For the biological and pharmaceutical activity of pyrimidinone derivatives, see: Modica *et al.* (2004 ▶); Panico *et al.* (2001 ▶). For related structures, see: Zheng *et al.* (2007 ▶); Hu *et al.* (2007 ▶); Xu (2008 ▶); Xu *et al.* (2006 ▶).
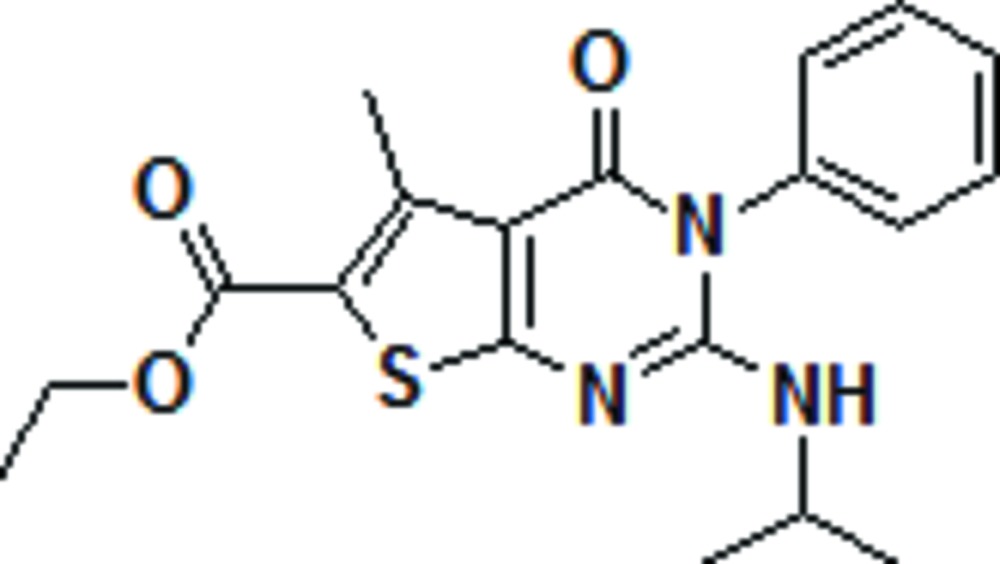


## Experimental

### 

#### Crystal data

C_19_H_21_N_3_O_3_S
*M* *_r_* = 371.45
Orthorhombic, 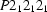

*a* = 8.5995 (14) Å
*b* = 13.673 (2) Å
*c* = 15.912 (3) Å
*V* = 1871.0 (5) Å^3^
*Z* = 4
Mo *K*α radiation
μ = 0.20 mm^−1^
*T* = 298 K
0.30 × 0.20 × 0.10 mm

#### Data collection

Bruker SMART 4K CCD area-detector diffractometer
Absorption correction: multi-scan (*SADABS*; Bruker, 2001 ▶) *T*_min_ = 0.943, *T*_max_ = 0.981
12706 measured reflections
4636 independent reflections
4149 reflections with *I* > 2σ(*I*)
*R*_int_ = 0.061

#### Refinement

*R*[*F* ^2^ > 2σ(*F* ^2^)] = 0.050
*wR*(*F* ^2^) = 0.123
*S* = 1.10
4636 reflections
271 parameters
6 restraints
H atoms treated by a mixture of independent and constrained refinement
Δρ_max_ = 0.33 e Å^−3^
Δρ_min_ = −0.22 e Å^−3^
Absolute structure: Flack (1983 ▶), 1984 Friedel pairs
Flack parameter: 0.09 (8)

### 

Data collection: *SMART* (Bruker, 2001 ▶); cell refinement: *SAINT-Plus* (Bruker, 2001 ▶); data reduction: *SAINT-Plus*; program(s) used to solve structure: *SHELXS97* (Sheldrick, 2008 ▶); program(s) used to refine structure: *SHELXL97* (Sheldrick, 2008 ▶); molecular graphics: *PLATON* (Spek, 2009 ▶); software used to prepare material for publication: *SHELXTL* (Sheldrick, 2008 ▶).

## Supplementary Material

Crystal structure: contains datablocks I, global. DOI: 10.1107/S1600536809045449/bt5115sup1.cif

Structure factors: contains datablocks I. DOI: 10.1107/S1600536809045449/bt5115Isup2.hkl

Additional supplementary materials:  crystallographic information; 3D view; checkCIF report

## Figures and Tables

**Table 1:** Hydrogen-bond geometry (Å, °)

*D*—H⋯*A*	*D*—H	H⋯*A*	*D*⋯*A*	*D*—H⋯*A*
C16—H16*A*⋯O2	0.96	2.33	3.015 (3)	128
C6—H6⋯O2^i^	0.93	2.58	3.479 (3)	164
N3—H3*A*⋯O1^ii^	0.77 (3)	2.28 (3)	2.949 (2)	145 (2)

Symmetry codes: (i) 
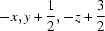
; (ii) 
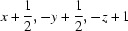
.

**Fig. 1.**
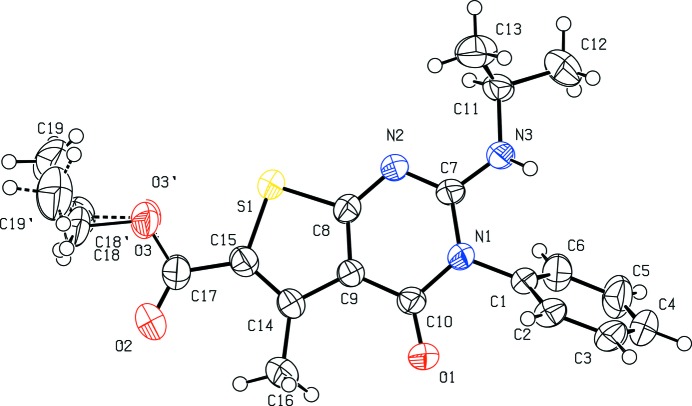
The molecular structure of the title compound, showing the atom-labeling scheme.

## supplementary crystallographic information

### Comment

Derivatives of pyrimidinone are attracting increasing attention in the synthetic chemistry community because of the important role played by such systems in many natural products, also in antibiotics and drugs (Modica *et al.*, 2004; Panico *et al.*, 2001). Recently, we have been interested in the synthesis of new thieno[3,2-*d*]pyrimidone derivatives. Some related X-ray crystal structure reports for pyrimidinone derivatives have been published (Zheng *et al.*, 2007; Hu *et al.* 2007; Xu *et al.*, 2006, 2008). Here, the structure of the title compound, which may be used as a new precursor for obtaining bioactive molecules, is reported (Fig. 1). In the molecule, the bond lengths and angles are unexceptional. The thienopyrimidinone rings are closer to coplanarity with maximum deviations 0.074 (2)Å for N3. The phenyl ring is twisted with respect to the pyrimidinone ring, with a dihedral angle of 66.30 (11)°. C18, C19 and attached hydrogen and O3 atoms are disordered over two sites, with refined occupancies of 0.537 (13) and 0.463 (13). Intramolecular C—H···O and intermolecular C—H···O, N—H···O hydrogen bonds interactions are present, which stabilize the conformation of the molecule and the crystal structure (Table 1).

### Experimental

To a solution of diethyl 5-((phenylimino)methyleneamino)- 3-methylthiophene-2,4-dicarboxylate(3 mmol) in anhydrous dichloromethane (15 ml) was added iso-propan-1-amine (3 mmol). After stirring the reaction mixture for 1 h, the solvent was removed and anhydrous ethanol (10 ml) with several drops of EtONa in EtOH was added. The mixture was stirred for 5 h at room temperature. The solution was concentrated under reduced pressure and the residue was recrystallized from ethanol to give the title compound in a yield of 85%. Crystals suitable for single-crystal X-ray diffraction were obtained by recrystallization from a mixed solvent of ethanol and dichloromethane (1:1 *v*/*v*) at room temperature.

### Refinement

All H-atoms bonded to C were positioned geometrically and refined using a riding model with C—H = 0.93 Å, *U*_iso_=1.2*U*_eq_ (C) for C*sp*^2^, C—H = 0.98 Å, *U*_iso_ = 1.2*U*_eq_ (C) for CH, C—H = 0.97 Å, *U*_iso_ = 1.2*U*_eq_ (C) for CH_2_, C—H = 0.96 Å, *U*_iso_ = 1.5*U*_eq_ (C) for CH_3_. The coordinates of the amino H atom were refined with *U*_iso_ = 1.2*U*_eq_(N). The terminal O-CH_2_CH_3_ moiety is disordered over two sites with refined occupancies of 0.537 (13) and 0.463 (13). The C-C bonds were restrained to 1.54 (1)Å, the C-O bonds to 1.45 (1)° and the O···C_methyl_ distances to 2.45 (2)Å.

### Crystal data

**Table:**

C_19_H_21_N_3_O_3_S	*F*(000) = 784
*M**_r_* = 371.45	*D*_x_ = 1.319 Mg m^−^^3^
Orthorhombic, *P*2_1_2_1_2_1_	Mo *K*α radiation, λ = 0.71073 Å
Hall symbol: P 2ac 2ab	Cell parameters from 4510 reflections
*a* = 8.5995 (14) Å	θ = 2.6–26.5°
*b* = 13.673 (2) Å	µ = 0.20 mm^−^^1^
*c* = 15.912 (3) Å	*T* = 298 K
*V* = 1871.0 (5) Å^3^	Block, colorless
*Z* = 4	0.30 × 0.20 × 0.10 mm

### Data collection

**Table:**

Bruker SMART 4K CCD area-detector diffractometer	4636 independent reflections
Radiation source: fine-focus sealed tube	4149 reflections with *I* > 2σ(*I*)
graphite	*R*_int_ = 0.061
φ and ω scans	θ_max_ = 28.3°, θ_min_ = 2.0°
Absorption correction: multi-scan (*SADABS*; Bruker, 2001)	*h* = −7→11
*T*_min_ = 0.943, *T*_max_ = 0.981	*k* = −18→17
12706 measured reflections	*l* = −21→18

### Refinement

**Table:**

Refinement on *F*^2^	Secondary atom site location: difference Fourier map
Least-squares matrix: full	Hydrogen site location: inferred from neighbouring sites
*R*[*F*^2^ > 2σ(*F*^2^)] = 0.050	H atoms treated by a mixture of independent and constrained refinement
*wR*(*F*^2^) = 0.123	*w* = 1/[σ^2^(*F*_o_^2^) + (0.0601*P*)^2^] where *P* = (*F*_o_^2^ + 2*F*_c_^2^)/3
*S* = 1.10	(Δ/σ)_max_ < 0.001
4636 reflections	Δρ_max_ = 0.33 e Å^−^^3^
271 parameters	Δρ_min_ = −0.21 e Å^−^^3^
6 restraints	Absolute structure: Flack (1983), 1984 Friedel pairs
Primary atom site location: structure-invariant direct methods	Flack parameter: 0.09 (8)

### Special details

**Table:**

Geometry. All e.s.d.'s (except the e.s.d. in the dihedral angle between two l.s. planes) are estimated using the full covariance matrix. The cell e.s.d.'s are taken into account individually in the estimation of e.s.d.'s in distances, angles and torsion angles; correlations between e.s.d.'s in cell parameters are only used when they are defined by crystal symmetry. An approximate (isotropic) treatment of cell e.s.d.'s is used for estimating e.s.d.'s involving l.s. planes.
Refinement. Refinement of *F*^2^ against ALL reflections. The weighted *R*-factor *wR* and goodness of fit *S* are based on *F*^2^, conventional *R*-factors *R* are based on *F*, with *F* set to zero for negative *F*^2^. The threshold expression of *F*^2^ > σ(*F*^2^) is used only for calculating *R*-factors(gt) *etc*. and is not relevant to the choice of reflections for refinement. *R*-factors based on *F*^2^ are statistically about twice as large as those based on *F*, and *R*- factors based on ALL data will be even larger.

### Fractional atomic coordinates and isotropic or equivalent isotropic displacement parameters (Å^2^)

**Table:**

	*x*	*y*	*z*	*U*_iso_*/*U*_eq_	Occ. (<1)
C1	0.2137 (2)	0.27871 (14)	0.47156 (12)	0.0406 (4)	
C2	0.2947 (3)	0.21627 (15)	0.41948 (14)	0.0488 (5)	
H2	0.3578	0.1675	0.4417	0.059*	
C3	0.2800 (3)	0.22783 (18)	0.33286 (15)	0.0606 (6)	
H3	0.3351	0.1869	0.2969	0.073*	
C4	0.1855 (4)	0.2987 (2)	0.30019 (16)	0.0694 (8)	
H4	0.1765	0.3056	0.2423	0.083*	
C5	0.1040 (4)	0.3595 (2)	0.35261 (17)	0.0716 (8)	
H5	0.0385	0.4070	0.3303	0.086*	
C6	0.1196 (3)	0.35009 (17)	0.43916 (15)	0.0552 (6)	
H6	0.0663	0.3921	0.4749	0.066*	
C7	0.2851 (2)	0.34531 (13)	0.61047 (13)	0.0395 (4)	
C8	0.2038 (2)	0.27158 (14)	0.72863 (12)	0.0408 (4)	
C9	0.1490 (2)	0.18802 (14)	0.68884 (12)	0.0376 (4)	
C10	0.1562 (2)	0.18457 (13)	0.59891 (13)	0.0391 (4)	
C11	0.4070 (3)	0.50790 (14)	0.60896 (14)	0.0464 (5)	
H11	0.3291	0.5270	0.6508	0.056*	
C12	0.4122 (4)	0.5855 (2)	0.5416 (2)	0.0834 (10)	
H12A	0.4823	0.5656	0.4979	0.125*	
H12B	0.4476	0.6460	0.5655	0.125*	
H12C	0.3101	0.5944	0.5185	0.125*	
C13	0.5612 (3)	0.4960 (2)	0.6528 (2)	0.0693 (7)	
H13A	0.5529	0.4459	0.6948	0.104*	
H13B	0.5899	0.5566	0.6790	0.104*	
H13C	0.6392	0.4779	0.6125	0.104*	
C14	0.0844 (2)	0.11758 (14)	0.74560 (13)	0.0400 (4)	
C15	0.0934 (2)	0.14955 (14)	0.82673 (14)	0.0446 (5)	
C16	0.0151 (3)	0.02200 (17)	0.71855 (17)	0.0597 (6)	
H16A	−0.0409	−0.0065	0.7647	0.089*	
H16B	0.0966	−0.0216	0.7012	0.089*	
H16C	−0.0547	0.0328	0.6724	0.089*	
C17	0.0542 (3)	0.09639 (19)	0.90376 (15)	0.0544 (6)	
C18'	0.0196 (9)	0.1295 (7)	1.0546 (4)	0.065 (2)	0.463 (13)
H18C	−0.0535	0.0755	1.0519	0.078*	0.463 (13)
H18D	−0.0262	0.1809	1.0885	0.078*	0.463 (13)
C19'	0.1698 (11)	0.0955 (10)	1.0941 (7)	0.099 (4)	0.463 (13)
H19D	0.2073	0.0388	1.0648	0.148*	0.463 (13)
H19E	0.1519	0.0792	1.1520	0.148*	0.463 (13)
H19F	0.2457	0.1468	1.0907	0.148*	0.463 (13)
O3'	0.0508 (11)	0.1657 (6)	0.9710 (4)	0.0593 (17)	0.463 (13)
C18	0.0891 (12)	0.0864 (5)	1.0496 (3)	0.070 (2)	0.537 (13)
H18A	0.1427	0.0240	1.0484	0.084*	0.537 (13)
H18B	−0.0205	0.0748	1.0601	0.084*	0.537 (13)
C19	0.1567 (16)	0.1515 (7)	1.1163 (5)	0.106 (3)	0.537 (13)
H19A	0.2569	0.1746	1.0984	0.160*	0.537 (13)
H19B	0.1676	0.1154	1.1676	0.160*	0.537 (13)
H19C	0.0889	0.2063	1.1253	0.160*	0.537 (13)
O3	0.1105 (10)	0.1383 (5)	0.9708 (3)	0.0599 (15)	0.537 (13)
N1	0.22489 (19)	0.26857 (11)	0.56226 (10)	0.0390 (4)	
N2	0.2725 (2)	0.34924 (12)	0.69284 (11)	0.0414 (4)	
N3	0.3553 (2)	0.41720 (12)	0.56877 (12)	0.0464 (4)	
H3A	0.387 (3)	0.4055 (18)	0.5245 (18)	0.056*	
O1	0.1063 (2)	0.11958 (11)	0.55348 (10)	0.0530 (4)	
O2	0.0060 (2)	0.01385 (13)	0.90784 (12)	0.0684 (5)	
S1	0.17644 (7)	0.26585 (4)	0.83568 (3)	0.05191 (16)	

### Atomic displacement parameters (Å^2^)

**Table:**

	*U*^11^	*U*^22^	*U*^33^	*U*^12^	*U*^13^	*U*^23^
C1	0.0506 (11)	0.0394 (10)	0.0320 (9)	−0.0100 (9)	0.0019 (8)	−0.0027 (8)
C2	0.0563 (12)	0.0467 (10)	0.0435 (11)	−0.0021 (9)	−0.0007 (9)	−0.0103 (9)
C3	0.0789 (16)	0.0605 (13)	0.0425 (12)	−0.0130 (12)	0.0112 (12)	−0.0177 (12)
C4	0.102 (2)	0.0714 (17)	0.0345 (12)	−0.0149 (17)	−0.0017 (13)	0.0018 (11)
C5	0.099 (2)	0.0705 (16)	0.0456 (15)	0.0111 (15)	−0.0045 (14)	0.0150 (13)
C6	0.0710 (15)	0.0511 (12)	0.0434 (13)	0.0092 (11)	0.0073 (11)	0.0047 (10)
C7	0.0475 (11)	0.0334 (9)	0.0375 (10)	−0.0033 (8)	0.0027 (8)	−0.0061 (7)
C8	0.0475 (11)	0.0422 (10)	0.0327 (9)	−0.0002 (9)	0.0015 (8)	−0.0026 (8)
C9	0.0428 (10)	0.0340 (8)	0.0359 (9)	−0.0008 (8)	−0.0028 (8)	0.0014 (7)
C10	0.0460 (10)	0.0325 (8)	0.0389 (10)	0.0009 (8)	−0.0033 (8)	−0.0008 (7)
C11	0.0578 (13)	0.0343 (9)	0.0472 (12)	−0.0073 (9)	0.0111 (10)	−0.0077 (8)
C12	0.122 (3)	0.0458 (13)	0.082 (2)	−0.0179 (16)	0.0038 (19)	0.0125 (14)
C13	0.0615 (15)	0.0708 (16)	0.0756 (19)	−0.0120 (13)	−0.0005 (14)	−0.0171 (15)
C14	0.0424 (10)	0.0358 (9)	0.0418 (11)	0.0007 (8)	0.0007 (8)	0.0052 (8)
C15	0.0475 (11)	0.0414 (10)	0.0449 (12)	−0.0033 (8)	−0.0007 (9)	0.0060 (9)
C16	0.0805 (17)	0.0412 (12)	0.0573 (15)	−0.0124 (11)	0.0060 (13)	0.0022 (11)
C17	0.0591 (14)	0.0637 (14)	0.0403 (12)	−0.0082 (11)	−0.0004 (10)	0.0081 (11)
C18'	0.082 (5)	0.059 (4)	0.053 (4)	0.003 (3)	0.020 (3)	0.003 (3)
C19'	0.099 (6)	0.128 (10)	0.070 (7)	0.009 (7)	−0.001 (5)	0.028 (7)
O3'	0.083 (5)	0.055 (4)	0.040 (2)	−0.004 (3)	0.006 (3)	0.007 (2)
C18	0.107 (6)	0.071 (4)	0.032 (3)	−0.005 (4)	−0.004 (3)	0.013 (3)
C19	0.193 (10)	0.073 (5)	0.054 (4)	−0.001 (5)	−0.024 (5)	0.003 (3)
O3	0.090 (4)	0.050 (3)	0.0398 (19)	0.000 (2)	−0.002 (2)	0.0111 (18)
N1	0.0515 (9)	0.0339 (7)	0.0315 (8)	−0.0024 (7)	0.0007 (7)	−0.0029 (7)
N2	0.0549 (10)	0.0348 (8)	0.0345 (9)	−0.0057 (7)	0.0030 (7)	−0.0040 (7)
N3	0.0623 (11)	0.0403 (8)	0.0365 (9)	−0.0117 (8)	0.0130 (8)	−0.0084 (7)
O1	0.0762 (11)	0.0409 (7)	0.0420 (9)	−0.0149 (7)	−0.0053 (8)	−0.0033 (6)
O2	0.0874 (13)	0.0600 (10)	0.0578 (11)	−0.0188 (10)	0.0060 (10)	0.0144 (9)
S1	0.0734 (4)	0.0499 (3)	0.0324 (2)	−0.0140 (3)	0.0024 (2)	−0.0019 (2)

### Geometric parameters (Å, °)

**Table:**

C1—C6	1.369 (3)	C13—H13A	0.9600
C1—C2	1.379 (3)	C13—H13B	0.9600
C1—N1	1.453 (2)	C13—H13C	0.9600
C2—C3	1.393 (3)	C14—C15	1.365 (3)
C2—H2	0.9300	C14—C16	1.499 (3)
C3—C4	1.367 (4)	C15—C17	1.464 (3)
C3—H3	0.9300	C15—S1	1.749 (2)
C4—C5	1.371 (4)	C16—H16A	0.9600
C4—H4	0.9300	C16—H16B	0.9600
C5—C6	1.390 (3)	C16—H16C	0.9600
C5—H5	0.9300	C17—O2	1.204 (3)
C6—H6	0.9300	C17—O3	1.303 (7)
C7—N2	1.316 (3)	C17—O3'	1.429 (8)
C7—N3	1.331 (3)	C18'—O3'	1.445 (7)
C7—N1	1.399 (2)	C18'—C19'	1.510 (8)
C8—N2	1.342 (2)	C18'—H18C	0.9700
C8—C9	1.389 (3)	C18'—H18D	0.9700
C8—S1	1.721 (2)	C19'—H19D	0.9600
C9—C14	1.433 (3)	C19'—H19E	0.9600
C9—C10	1.433 (3)	C19'—H19F	0.9600
C10—O1	1.223 (2)	C18—O3	1.453 (6)
C10—N1	1.417 (2)	C18—C19	1.502 (7)
C11—N3	1.464 (3)	C18—H18A	0.9700
C11—C13	1.508 (4)	C18—H18B	0.9700
C11—C12	1.509 (4)	C19—H19A	0.9600
C11—H11	0.9800	C19—H19B	0.9600
C12—H12A	0.9600	C19—H19C	0.9600
C12—H12B	0.9600	N3—H3A	0.77 (3)
C12—H12C	0.9600		
			
C6—C1—C2	120.9 (2)	H13B—C13—H13C	109.5
C6—C1—N1	118.77 (19)	C15—C14—C9	111.03 (18)
C2—C1—N1	120.30 (19)	C15—C14—C16	125.0 (2)
C1—C2—C3	118.6 (2)	C9—C14—C16	123.99 (19)
C1—C2—H2	120.7	C14—C15—C17	128.3 (2)
C3—C2—H2	120.7	C14—C15—S1	113.04 (16)
C4—C3—C2	120.7 (2)	C17—C15—S1	118.50 (17)
C4—C3—H3	119.7	C14—C16—H16A	109.5
C2—C3—H3	119.7	C14—C16—H16B	109.5
C3—C4—C5	120.2 (2)	H16A—C16—H16B	109.5
C3—C4—H4	119.9	C14—C16—H16C	109.5
C5—C4—H4	119.9	H16A—C16—H16C	109.5
C4—C5—C6	119.8 (2)	H16B—C16—H16C	109.5
C4—C5—H5	120.1	O2—C17—O3	119.7 (3)
C6—C5—H5	120.1	O2—C17—O3'	125.0 (3)
C1—C6—C5	119.8 (2)	O3—C17—O3'	26.4 (3)
C1—C6—H6	120.1	O2—C17—C15	126.1 (2)
C5—C6—H6	120.1	O3—C17—C15	112.4 (3)
N2—C7—N3	120.23 (18)	O3'—C17—C15	107.6 (3)
N2—C7—N1	123.12 (18)	O3'—C18'—C19'	109.3 (6)
N3—C7—N1	116.64 (17)	O3'—C18'—H18C	109.8
N2—C8—C9	127.35 (18)	C19'—C18'—H18C	109.8
N2—C8—S1	121.07 (14)	O3'—C18'—H18D	109.8
C9—C8—S1	111.57 (14)	C19'—C18'—H18D	109.8
C8—C9—C14	113.40 (18)	H18C—C18'—H18D	108.3
C8—C9—C10	117.90 (18)	C17—O3'—C18'	117.8 (5)
C14—C9—C10	128.62 (19)	O3—C18—C19	105.8 (5)
O1—C10—N1	119.46 (18)	O3—C18—H18A	110.6
O1—C10—C9	126.79 (19)	C19—C18—H18A	110.6
N1—C10—C9	113.72 (17)	O3—C18—H18B	110.6
N3—C11—C13	112.19 (19)	C19—C18—H18B	110.6
N3—C11—C12	107.1 (2)	H18A—C18—H18B	108.7
C13—C11—C12	112.2 (2)	C18—C19—H19A	109.5
N3—C11—H11	108.4	C18—C19—H19B	109.5
C13—C11—H11	108.4	H19A—C19—H19B	109.5
C12—C11—H11	108.4	C18—C19—H19C	109.5
C11—C12—H12A	109.5	H19A—C19—H19C	109.5
C11—C12—H12B	109.5	H19B—C19—H19C	109.5
H12A—C12—H12B	109.5	C17—O3—C18	116.4 (5)
C11—C12—H12C	109.5	C7—N1—C10	122.44 (16)
H12A—C12—H12C	109.5	C7—N1—C1	119.84 (16)
H12B—C12—H12C	109.5	C10—N1—C1	117.29 (15)
C11—C13—H13A	109.5	C7—N2—C8	115.24 (17)
C11—C13—H13B	109.5	C7—N3—C11	123.06 (18)
H13A—C13—H13B	109.5	C7—N3—H3A	117.6 (19)
C11—C13—H13C	109.5	C11—N3—H3A	117.7 (19)
H13A—C13—H13C	109.5	C8—S1—C15	90.95 (10)
			
C6—C1—C2—C3	0.6 (3)	O3—C17—O3'—C18'	−72.8 (10)
N1—C1—C2—C3	179.51 (18)	C15—C17—O3'—C18'	−177.9 (5)
C1—C2—C3—C4	−1.0 (4)	C19'—C18'—O3'—C17	85.6 (13)
C2—C3—C4—C5	0.1 (4)	O2—C17—O3—C18	−9.1 (7)
C3—C4—C5—C6	1.0 (5)	O3'—C17—O3—C18	100.6 (13)
C2—C1—C6—C5	0.5 (4)	C15—C17—O3—C18	−174.9 (5)
N1—C1—C6—C5	−178.4 (2)	C19—C18—O3—C17	−177.4 (12)
C4—C5—C6—C1	−1.4 (4)	N2—C7—N1—C10	−5.2 (3)
N2—C8—C9—C14	178.76 (19)	N3—C7—N1—C10	175.90 (18)
S1—C8—C9—C14	−0.3 (2)	N2—C7—N1—C1	167.00 (19)
N2—C8—C9—C10	−4.3 (3)	N3—C7—N1—C1	−11.9 (3)
S1—C8—C9—C10	176.67 (15)	O1—C10—N1—C7	−179.60 (19)
C8—C9—C10—O1	−176.0 (2)	C9—C10—N1—C7	2.4 (3)
C14—C9—C10—O1	0.5 (4)	O1—C10—N1—C1	8.0 (3)
C8—C9—C10—N1	1.9 (3)	C9—C10—N1—C1	−170.04 (17)
C14—C9—C10—N1	178.34 (18)	C6—C1—N1—C7	−62.7 (3)
C8—C9—C14—C15	−0.6 (3)	C2—C1—N1—C7	118.4 (2)
C10—C9—C14—C15	−177.1 (2)	C6—C1—N1—C10	109.9 (2)
C8—C9—C14—C16	179.0 (2)	C2—C1—N1—C10	−69.0 (3)
C10—C9—C14—C16	2.4 (4)	N3—C7—N2—C8	−178.13 (19)
C9—C14—C15—C17	−173.8 (2)	N1—C7—N2—C8	3.0 (3)
C16—C14—C15—C17	6.7 (4)	C9—C8—N2—C7	1.7 (3)
C9—C14—C15—S1	1.2 (2)	S1—C8—N2—C7	−179.30 (16)
C16—C14—C15—S1	−178.38 (19)	N2—C7—N3—C11	−6.7 (3)
C14—C15—C17—O2	−0.3 (4)	N1—C7—N3—C11	172.24 (19)
S1—C15—C17—O2	−175.0 (2)	C13—C11—N3—C7	81.3 (3)
C14—C15—C17—O3	164.5 (5)	C12—C11—N3—C7	−155.1 (2)
S1—C15—C17—O3	−10.3 (5)	N2—C8—S1—C15	−178.33 (18)
C14—C15—C17—O3'	−167.9 (5)	C9—C8—S1—C15	0.79 (16)
S1—C15—C17—O3'	17.4 (5)	C14—C15—S1—C8	−1.14 (17)
O2—C17—O3'—C18'	14.3 (9)	C17—C15—S1—C8	174.36 (18)

### Hydrogen-bond geometry (Å, °)

**Table:**

*D*—H···*A*	*D*—H	H···*A*	*D*···*A*	*D*—H···*A*
C16—H16A···O2	0.96	2.33	3.015 (3)	128
C6—H6···O2^i^	0.93	2.58	3.479 (3)	164
N3—H3A···O1^ii^	0.77 (3)	2.28 (3)	2.949 (2)	145 (2)

Symmetry codes: (i) −*x*, *y*+1/2, −*z*+3/2; (ii) *x*+1/2, −*y*+1/2, −*z*+1.
